# Herbal Medicine in Mexico: A Cause of Hepatotoxicity. A Critical Review

**DOI:** 10.3390/ijms17020235

**Published:** 2016-02-15

**Authors:** Bárbara Valdivia-Correa, Cristina Gómez-Gutiérrez, Misael Uribe, Nahum Méndez-Sánchez

**Affiliations:** Liver Research Unit, Medica Sur Clinic & Foundation, 14050 Mexico City, Mexico; bisba100@hotmail.com (B.V.-C.); cristinacgg@hotmail.com (C.G.-G.); muribe@medicasur.org.mx (M.U.)

**Keywords:** herbal medicine, herb-induced liver injury (HILI), drug-induced liver injury (DILI), hepatotoxicity, herbal drug supplements (HDS), adverse events, regulation

## Abstract

In Mexico, herbal products are commonly used as therapeutic tools. The analysis of several publications reveals that there are dozens of different herbs and herbal products used for different reasons, some of which have been implicated in causing toxic liver disease. However, methodological aspects limit the attribution of causality, and the precise incidence and clinical manifestations of herb-induced liver injury have not been well characterized. This review outlines the history of traditional herbal medicine in Mexico, critically summarizes the mechanisms and adverse effects of commonly used herbal plants, and examines the regulatory issues regarding the legal use of these products.

## 1. Introduction

Herbal remedies are therapeutic products and foods made from the leaves, seeds, flowers, and roots of plants, or from extracts thereof [1]. They have for centuries constituted an important element in the treatment of several diseases. The true prevalence of herbal use is unknown because reporting rates vary significantly and a large number of patients do not disclose their use [2]. Nevertheless, the use of herbal remedies has increased worldwide over recent years [3]. These are presented in many forms, and crude herbal preparations are more prevalent in developing countries, which makes their use more difficult to regulate.

## 2. History

Multiple manuscripts about the indigenous populations that were written during the sixteenth century highlight the importance of herbal medicine in Mexico’s history. One of the most important examples is the Badianus manuscript, which includes numerous colored illustrations of medicinal plants. The later Historia Natural de la Nueva España (Natural History of New Spain) gathered information not only about medicinal plants but also about the animals and minerals of the New World. During the 1990s, ethnomedicinal research began to develop, combining ethnopharmacy and ethnobotany as the principal avenues of study. Many publications in Mexico and Latin America started to explore the use of medicinal plants by modern populations. In 1994, Aguilar *et al.* [4] created the first Mexican herbarium [5]. Currently, digital libraries provide a fundamental resource for the investigation of traditional medicine in Mexico; they incorporate approximately 1045 monographs about medicinal plants.

## 3. Epidemiology and Geographic Distribution in Mexico

Despite the growing evidence from publications describing herb-induced liver injury (HILI), determining its true incidence remains a challenge, mainly because of the absence of regulatory guidelines and legal controls. After China, with almost 5000 recorded medicinal plant species, Mexico has the second highest number with 4500 recorded species [6]. Notably, reports indicate that the Chinese population has the highest number of HILI cases. Current estimates suggest that herbs cause 15% of drug-induced liver injury (DILI). Further, a recent tabular compilation of 77 publications established causality for 28 of 57 different herbs and herbal mixtures in this population [7].

**Figure 1:**
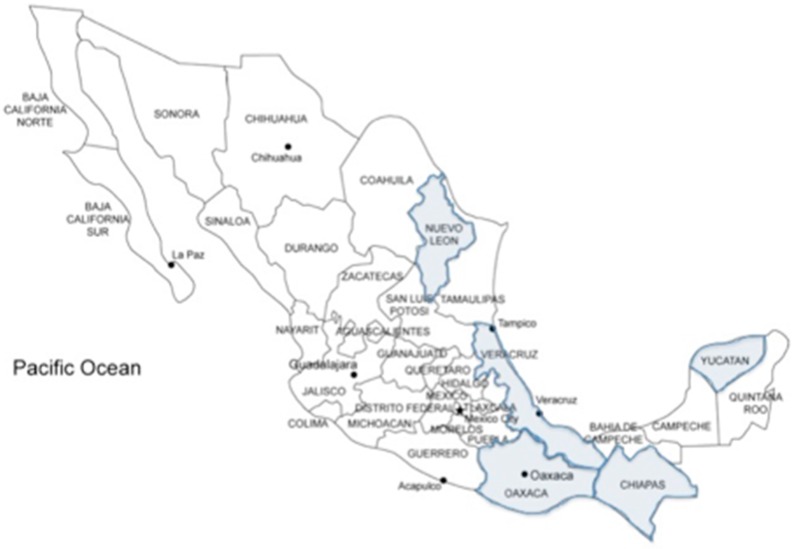
Epidemiological distribution of knowledge about herbal medicinal plants in Mexico highlighting that fieldwork to establish the used of medicinal plant species has been carried out only in Nuevo Leon, Veracruz, Oaxaca, Chiapas, and Yucatan states.

In Mexico, the distribution of herbal medicine correlates well with the regions inhabited by indigenous communities. There are few studies that determine the distribution of medicinal plant knowledge in Mexico. Most of the fieldwork has been done in Oaxaca, Veracruz, Nuevo León, Yucatán, and Chiapas, areas with a large proportion of indigenous inhabitants. Estrada *et al.* [8] collected a total of 163 medicinal plant species used in the southern area of Nuevo León. People use 5% of the total state flora as medicinal plants, and 235 different medicinal uses were recorded. Rural communities dominate the state of Oaxaca; approximately 15 different indigenous groups make up 87% of the population in Sierra Norte. Giovannini *et al.* [9] conducted a field study in a community of about 400 people in Paloma Alta, Oaxaca. In this area, Mazatec medicine is quite popular. Structured interviews provided detailed information about the species used. The respondents recalled 82 species used medicinally. Of these, only 39 were mentioned more than once, the four most common being *Cestrum nocturnum*, *Psidium guajava*, *Aristolochia odoratissima,* and *Zingiber officinale.* Self-treatment is the most common first therapeutic choice in this area. Frei *et al.* [10] studied Zapotec medicinal botanic diversity, and documented 445 different medicinal plant species. Dermatological conditions and gastrointestinal disorders were the most frequently cited medical problems for which these were used. Finally, Ankli *et al.* [11], as part of an ethnopharmacological field study in the Yucatan Mayan Peninsula, evaluated 48 medicinal plants using several biological assays. This was one of the first studies that obtained information regarding the phytochemically and pharmacologically active properties of the plants (Figure 1).

Despite the efforts made to determine the different types of herbal remedies used in Mexico, there are only case reports and some series describing HILI in this country. There is insufficient quantitative data about the consumption of herbs, the number of HILI patients, and an approximation of the population at risk to allow appropriate assessment of the risks from a specific herbal product [3]. Most of the information is extrapolated from the United States (US), where the estimated incidence of HILI has increased almost threefold over the past decade [12]. A 2008 review found that approximately one in five adults in the US reported taking at least one herbal product [2]. It is likely that in Mexico the rise is exponential. The only study that has determined the incidence of hepatic adverse effects with these products was conducted at a German hospital for traditional medicine and reported an approximately 1% incidence of HILI defined by alanine aminotransferase ALT elevation after consumption of Chinese herbal drugs [13].

## 4. Herb-Induced Liver Injury Overview

### 4.1. General Aspects

Liver injury caused by herbal products is an important but disregarded subject. There is a tendency toward increasing the use of herbal products and supplements not only for medical reasons but also for weight control and even bodybuilding, which represents a lucrative business in Europe and the US. For most herbs, HILI is a rare complication that displays several characteristics similar to DILI. Therefore, most of the information about herbal medicine-related hepatotoxicity emerges from studies of DILI. However, many individuals believe that herbal medicines are harmless and represent a more “natural” means of managing or preventing disease. Moreover, marketing regulations for herbal medicines are less strict than those for conventional drugs, leading to an abundance of easily accessible herbal medicine products [14].

### 4.2. Classification

HILI represents the second most common cause of DILI in Western countries. In Mexico there is not a proper classification of herbal medicines, although there have been several efforts to standardize herbal products. In 1986, the Unit for Research into Traditional Medicine and Drug Development of the Mexican Social Security Institute created a dictionary of traditional Mexican medicine. The main objective was to compile information on human resources, causes of demand, and treatment plans involving traditional medicine in rural areas since 1979. In 1989, the project was implemented with the National Indigenous Institute. Between 1990 and 1994, the National Autonomous University of Mexico decided to prepare a new version, using the advantages of information technology and communications. In this Digital Library of Traditional Mexican Medicine are seven herbal products with warnings about hepatotoxicity: *Scoparia dulcis* L. (maidenhair), *Citrus aurantium* L. (citrus orange), *Prunus persica* L. (peach), *Rosmarinus officinalis* L. (rosemary), *Equisetum hyemale* L. (horse tail), *Tilia mexicana* Schlechtendal (tilia), and *Morus alba* L. (white mulberry). Most of these products are used daily by the Mexican population [5].

### 4.3. Risk Factors

It is important to consider several risk factors for HILI, including the components of herbs and their side effects, together with the chemical products used on the crop, the environment, and clinical host-related risk factors. Using this information it is easier to determine which herbal remedies might induce direct toxicity. The side effects may also occur because of contaminants in the herbal products, because heavy metals including lead, mercury or arsenic and other undeclared chemicals are added to the herbs to produce a desired effect. The plants synthesized a vast collection of secondary metabolites as defense mechanisms to protect themselves against infections from pathogens including bacteria, fungi, and viruses [15]. The clinical host-related risk factors include age and gender as nonmodifiable risk factors. In some studies, those patients with liver injury attributed to bodybuilding herbal drug supplements (HDS) were male, younger, and with fewer comorbid conditions compared with those using nonbodybuilding HDS who were female, older, and with a higher incidence of comorbid conditions such as diabetes and neurological, heart, pulmonary, and gastrointestinal diseases. Our understanding of environmental factors is limited; some proposed factors are coffee, alcohol consumption, and diet. Navarro *et al.* [16] showed that alcohol consumption was more highly associated with side effects in the group taking bodybuilding supplements compared with the groups taking other medications (79% *vs.* 54% *vs.* 48%, respectively; *p* < 0.001). More information and further studies are needed to determine the direct relationship between these factors and liver injury.

### 4.4. Clinical Course

The clinical presentation of HILI varies from asymptomatic or abnormal hepatic biochemical tests to acute liver failure requiring a liver transplant. The initial manifestations begin with nonspecific constitutional symptoms followed by jaundice [17]; Chalasani *et al.* [18] described 28 patients with DILI in the US DILI Network database. Fifty percent of these patients were female, the mean age was 45 years, hepatocellular injury was the most common side effect present in 63% of the patients, and cholestatic injury was present in 17%; 88% of cases of DILI were of mild to moderate severity and the rest were severe or fatal; 3.5% of patients required a liver transplant and 9% developed chronic DILI. Suk *et al.* [19] performed a prospective nationwide study of DILI in South Korea. A total of 371 cases were reported; the majority of patients were female (63.3%) and the median age was 49 (16–79) years; the causes included herbal medications in 27.5% of cases, prescription or nonprescription medications in 27.3%, health foods or dietary supplements in 13.7%, medicinal herbs or plants in 9.4%, folk remedies in 8.6%, and herbal preparations in 3.2%. Only nine cases were linked to acetaminophen. The pattern of disease was hepatocellular in 76.3% of cases, mixed in 14.8%, and cholestatic in 8.9%. Five patients died or underwent transplantation. An herbal decoction (*n* = 191) was found to be the most common etiology. Among medications, an antifungal agent was found to be the most common cause of DILI.

In 2014, Navarro *et al.* [16] described a prospective study between 2004 and 2013 to characterize hepatotoxicity and its outcomes, comparing HDS *versus* medications. The study took place in eight US referral centers that are part of the Drug-Induced Liver Injury Network (DILIN). The final sample was 130 patients (15.5% of the 839 subjects enrolled). Of these, 45 had injury caused by bodybuilding HDS, 85 had injury caused by nonbodybuilding HDS, and 709 had injury caused by medications. The incidence of liver injury caused by HDS increased from 7% to 20% (*p* < 0.001) during the study period. This increase involved both bodybuilding HDS (from 2% in 2004–2005 to 8% in 2010–2012; *p* < 0.007) and nonbodybuilding HDS (from 5% in 2004–2005 to 12% in 2010–2012; *p* < 0.05). In laboratory tests, the patients with injury from nonbodybuilding HDS had the highest mean ALT (1019 U/L) and AST (815 U/L) values and intermediate mean ALP (212 U/L) and bilirubin levels (7.5 mg/dL). Patients with medication-induced injury had intermediate ALT and AST elevations (505 and 319, respectively), and the highest ALP and lowest bilirubin levels. Bodybuilding HDS caused prolonged jaundice with a median of 91 days in young men, but did not result in any fatalities or liver transplants. The HDS cases presented as hepatocellular injury, predominantly in middle-aged women. Liver transplant was required more frequently among patients with injury from nonbodybuilding HDS than in those with hepatotoxicity from conventional medications (13% *vs.* 3%, respectively, *p* < 0.001). The mean age of transplant recipients was 56 years (range, 27–73), all 13 were female, and 9 (69%) were white. Not surprisingly, patients with more severe hepatocellular injury (*R* value > 5) progressed to liver transplant more quickly than did those with cholestatic/mixed liver injury (median range) days from onset to death/transplant was 28 (2–77) in the hepatocellular group *vs.* 234 (61–263) in the cholestatic/mixed group; *p* < 0.004). The group taking bodybuilding HDS also had fewer comorbid conditions. Diabetes and neurological, heart, pulmonary, and gastrointestinal disease were more common among patients with conventional medication-associated liver injury. The clinical progress of patients with hepatotoxicity from bodybuilding HDS had a more protracted course than did the conventional medicine group [16].

The liver injury diagnosis begins with a history of the ingested HDS and exclusion of other causes of injury, such as viral hepatitis, autoimmune disease, anatomic malformations, and metabolic disturbance and in particular, hepatitis E virus (HEV) [20,21]. The diagnosis of DILI caused by HDS is difficult, because none of the conventional assessment approaches accurately assesses HDS-associated liver injury [20]. Because of the nonspecific clinical features of this disease, a good medical history that takes into account the consumption of herbal products and supplements and a physical examination is required, together with liver function tests and an awareness of the existence of HILI and differential diagnoses.

### 4.5. Pathogenic Aspects of HILI

**Figure 2:**
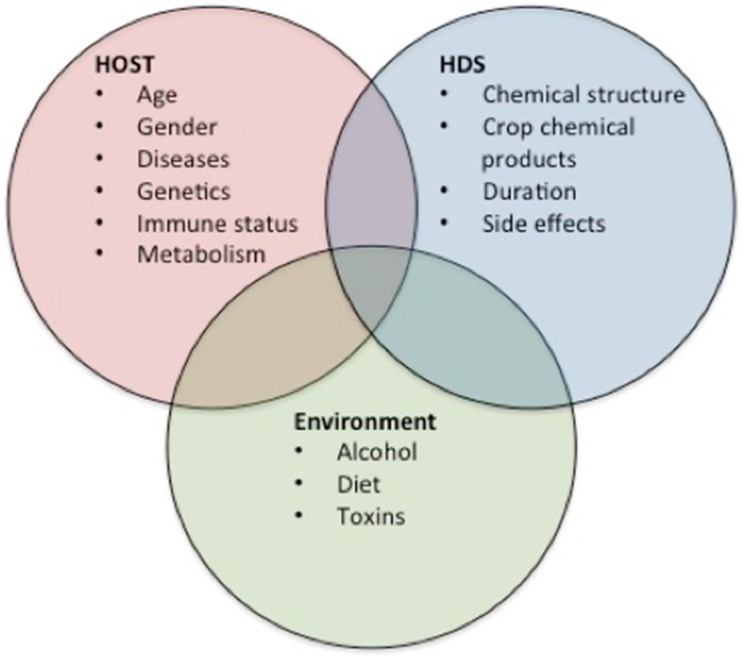
HILI Pathogenesis. This figure illustrates the various theories of the pathogenesis of herb-induced liver injury (HILI). Most of these overlap with those for drug-induced liver injury (DILI).

There are several theories about the pathophysiology of liver injury, the majority of which are based on DILI. The strong and consistent association of DILI susceptibility with various single nucleotide polymorphisms in the human leukocyte antigen (HLA) region suggests that the host immune response plays a key role in DILI pathogenesis. Polymorphisms in HLA genes mostly map to the antigen-binding cleft, which allows diversification of the repertoire of self-derived and pathogen-derived peptide antigens to be presented to T cells [22]. The “damage hypothesis” suggests that the inadvertent generation of reactive metabolites or parent drug-protein complexes can directly or indirectly mediate intracellular damage *via* oxidative endoplasmic reticulum stress, mitochondrial damage, or inhibition of the bile salt export pump. The “hapten hypothesis” suggests that the drug-protein or metabolite-protein adducts lead to inadvertent activation of the adaptive immune system (Figure 2) [7].

## 5. Traditional Herbal Products Used in Mexico

Herbs commonly used in Mexico that have hepatotoxic effects are listed in Table 1.

**Table 1:** Compilation of herbs and herbal products commonly used in Mexico. This table illustrates examples of medicinal plants commonly used in Mexico, their popular names, cultural uses, and active compounds. It showcases the complexity of herbal products and the challenging facets of their toxicity.

Medical Plant	Popular Names	Cultural Uses	Active Components	Side Effects
*Scoparia dulcis* L. (Maidenhair) [4]	Anisillo, candejilla, epazote bush, epazote sea grass blow, lentejilla; Michoacan: mishishe (Nahua); Nayarit: golpesal (Heart); Oaxaca: nax podeey (Mixe).	Diarrhea and stomach pain, toothache	Flavonoids apigenin, deflavona hexahydroxy -glucuronide, himenoxín, linarín, luteolin, glycoside; saponaretín, escutelarín, methyl ester, vitexin vicenín; dulcinol diterpenes, escopaduline acids A and B, escopaduline, escopariol; α-amyrin triterpenes, betulinic acid, dulcioico, friedelin	Hepatotoxicity
*Citrus aurantium* L. (Citrus orange) [4]	Orange, orange leaves, lemon, monument, orange, sour orange, sour orange, orange doghouse, orange Castile, cucho orange, orange, sour orange, bitter orange. Morelos: naranjaxocotl; Oaxaca: cajel, ma ji gui ruu, suuikh, tsuiky; Puebla: chichicarajas (Nahua), ixcapehto (Otomi), skeja Lasus (Totonac), Skaja laxux, xocot; Quintana Roo: cituhuk, pakal dzut, suut’spak‘aal (Maya); Veracruz: tsotso; San Luis Potosi; tdimalon lanash, kaxiy lanash (tenek)	Dysentery, stomach pain, abortion, asthma, colds, fever	Monoterpenes camphene, geraniol, limonene and linalool; methyl anthranilate and Citra, flavonoids, Rhamno-glycosides hesperidin, hesperidin glycosides, phenols; the sterols campesterol and β-sitosterol and carotenoid α-tocophero	Hepatotoxicity Teratogenicity
*Prunus persica* L. (Peach) [4]	San Luis Potosí: tulasnu (tenek)	Antiparasitic, dysentery, stomach pain	Epigalato of catechin, the diglucosides of kaempferol and quercetin, and the sterol β-sitosterol	Hepatotoxicity
*Rosmarinus officinalis* L. (Rosemary) [4,23]	Michoacan: romeru	Stomach pain, biliary colic, cold	1,8-cineol monoterpenes, bornyl acetate, camphor, α- and β-pinene, α-terpineol, nerol, geraniol, eugenol, nerolidol, slime-Neno, verbenol, myrcene	Hepatotoxicity
*Equisetum hyemale* L. (Horse tail) [4,23]	Carricillo. Estado de México: carrizo (mazahua); Michoacán: k uture (purhépecha); Sonora: cab’ bager (pima)	Abdominal pain, urinary tract infections	Carotenoids α- and β-carotene, lutein epoxide, licofíl, violaxantín and zeaxanthin	Hepatotoxicity
*Tilia mexicana Schlechtendal* (Tilia) [4]	Flordetila, flordetilia, tilia; Michoacán: sirimo (purhépecha), cirimo	Calm the nerves, menstrual pain	*p*-Coumaric acid, kaempferol, quercetin and terpenoid Constituents. Volatile oils, Including citral, citronellal, citronellol, eugenol, and limonene	Hepatotoxicity
*Morus alba* L. (White Mulberry) [4,23]	Moral, mora blanca, mora de tierra caliente, moran, moran hembra; Puebla: kimucucuk kiui (totonaco)	Muscle pain, respiratory diseases	Monoterpene geraniol limonene, linalool, acetate and α-pinene	Hepatotoxicity
*Opuntia ficusindica* (Nopal) [15,24]	Nopal, Cactus	Diabetes and others	Fibrous polysaccharide (fiber) and pectin	Diarrhea, nausea, abdominal fullness and headache
*Mentha piperita* (Peppermint) [15,24]	Mint	Gastrointestinal tract ailments and others	Acetaldehyde, amyl alcohol, menthyl esters, limone, pinene, phellandrene, cadinene, pugelone, and dimethyl sulfide, α-pinene, sabinene, terpinolene, ocimene, γ-terpinene, fenchene, α- and β-thujone, citronellol	Hepatotoxicity
*Larrea divaricate* (Chaparral) [15,25]	Governor, Creosote Bush, Greasewood, Hediondilla, Larreastat	Arthritis and others	Nordihydroguaiaretic acid	Hepatotoxicity
*Taraxacum officinale* (Dandelion) [15,24]	Blowball dandelion, cankerwort, Common Dandelion, Dudal, Herba Taraxaci, Lion‘s Tooth	Hepatic and biliary ailments, viral and bacterial infections, cancer and others	Quercetin, luteolin, luteolin-7-*O*-gluccoside, *p*-hydroxyphenylacetic acid, germacranolide acids, chlorogenic acid, chicoric acid, and monocaffeyltartaric acid, scopoletin, aesculetin, aesculin, cichoriin, arnidiol, and faradiol, caffeic acid, taraxacoside, taraxasterol, inulin and high potassium content	Allergic reactions, palpitations, syncope and erythema multiform
*Verbascum densiflorum* (Mullein) [15,24]	Mullein, Aaron's Rod, Adam's Flannel, American Mullein, Orange Mullein, Rag Paper	Inflammatory ailments in respiratory tract and others	Harpagoside, harpagide, aucubin, hesperidin, verbascoside, saponins, and volatile oils	None reported
*Matricaria recutita* (Chamomile) [15,24]	Chamomile, Blue Chamomile, Camomilla, Camomille Allemande, Cham, Echte Kamille, Fleur de Camomile	Gastrointestinal tract ailments and others	Quercetin, apigenin, and coumarins, and the essential oils matricin, chamazulene, α bisaboloid, and bisaboloid oxides	Allergic reactions and conjunctivitis
*Passiflora incarnate* (Passion flower) [15,24]	Crown of Christ, Passion Flower, Madre Selva, Passionflower, Passiflore, passiflorine, Passionaria	Insomnia, and anxiety or nervousness	Flavonoids apigenin, luteolin, quercetin, kaempferol, and vitexin, harmine, harmaline, harmalol, harman, and harmin. Other constituents include maltol and ethyl maltol	Dizziness, confusion and ataxia; Vasculitis; nausea, vomiting, drowsiness, tachycardia; hepatic and pancreatic toxicity
*Aloe vera* (Aloe) [15,24]	Zabila, Aloe Vera, Aloe Latex, Aloe Perfoliata, Burn Plant, Elephant‘s Gall, Gvarapatha, Gvar Patha, Indian Aloe, and others	Gastrointestinal ailments, wound healing and others	Emodin anthrone, dithranol, chrysarobin, carboxypeptidase, magnesium lactate, C-glucosyl chromone, salicylate and allantoin. Aloe latex belongs to the anthraquinone family and contains a tricyclic anthracene nucleus	Decrease platelet aggregation; Prolong bleeding time; Diarrhea and loss of water and electrolytes

## 6. Regulation of Herbal Products and Social Implications

Despite the ancestral use of traditional medicines by different populations, it was only very recently that a legal process began to assure their recognition within a legal framework. However, the precedent for regulation is difficult to establish. The major legislative tool was created in 1974, when the World Health Organization (WHO) urged developing countries to employ herbal medicine to “achieve the needs that had not been accomplished with modern therapies”. In 1993, the Food and Drug Administration (FDA) examined herbal supplements and products and created the Dietary Supplement Health and Education Act (DSHEA) [26]. In 2007, it launched the “Final Rule: Current Good Manufacturing Practice in Manufacturing, Packing, Labeling or Holding Operations for Dietary Supplements” [2].

Between 1994 and 1998, the Ministry of Health in Mexico established a Mexican classification of traditional medicine with the purpose of creating a systematic review according to the legal framework [2]. Subsequently, and in an attempt to standardize these products, an Official Norm was enacted in 2013, which regulates the usage and packaging of herbal remedies [27]. Nevertheless, legal coverage is undefined in some parts of the country.

The principal problem that faces regulation is that the precautions for the use of herbal medicines can be confusing for some because these products are considered “naturally occurring” rather than “man-made” compounds [1]. Moreover, they are available in various forms, including teas, capsules, and tablets, without prescription from a health care professional. In relation to herbal product quality, the misidentification of plants and the presence of contaminants, impurities, and adulterants still remain key problems [3].

There are some concerns regarding the use of herbal remedies, and few studies have determined the actual usage of these products within the Mexican population. It is estimated that 85% of medical practitioners have become acquainted with herbal medicines and almost 75% have recommended their use. In contrast, 92% of patients accepted and had knowledge of herbal medicines and 90% used them regularly [28]. A recent econometric model found a significant positive association between individual knowledge of medicinal plants and individual knowledge of pharmaceuticals, demonstrating that these coexist in a way that might be interpreted as complementary [9]. This idea indicates that ethnobotanical knowledge continues to be important for treating illness in many rural communities, despite the growing access to health care clinics and pharmaceuticals [29]. Consistent with this, Latino immigrants in the US experience significant impediments to accessing health care, forcing them to seek alternative treatments. A recent study demonstrated that health care barriers, cultural norms, self-care, and self-prescription were the four domains that influenced the use of alternative medicine [30]. Brown *et al.* [31] found that in the Texas–Mexico border cities, 68.3% of pharmacists recalled having patients using complementary and alternative medicine, with 39.4% of them presenting with medicine-related problems.

## 7. Conclusions and Future Challenges

In summary, herbal medicines are widely used in developing countries, and it will not be possible in the near future to scientifically evaluate all the specimens. However, although these herbal medicines are perceived to be safe some can cause severe liver injury. The exact incidence of HILI is difficult to determine and is probably underreported. Therefore, from a pharmacological perspective, a careful separation of products into their multiple components together with ethnopharmacological and toxicological studies are needed to determine the most important taxa and their adverse effects. From a clinical point of view, it is critical that we move forward in the recognition of signs and symptoms indicative of disturbances in liver function, since a potential differential diagnosis of acute liver failure can improve the prognosis in these patients. Education of patients and physicians is essential, particularly regarding clarification and emphasis that “not all natural products are safe”. These elements can change the perspective of the problem.
